# *In vitro-in vivo* availability of metformin hydrochloride-PLGA nanoparticles in diabetic rats in a periodontal disease experimental model

**DOI:** 10.1080/13880209.2021.2002369

**Published:** 2021-11-22

**Authors:** Aline de Sousa Barbosa Freitas Pereira, Maria Laura de Souza Lima, Arnobio Antonio da Silva-Junior, Emanuell dos Santos Silva, Raimundo Fernandes de Araújo Júnior, Agnes Andrade Martins, Jovelina Samara Ferreira Alves, Artur de Santana Oliveira, Leandro De Santis Ferreira, Emily Cintia Tossi de Araújo Costa, Gerlane Coelho Bernardo Guerra, Caroline Addison Carvalho Xavier de Medeiros, Gerly A. C. Brito, Renata Ferreira de Carvalho Leitao, Aurigena Antunes de Araújo

**Affiliations:** a Post-Graduation Program in Oral Science, Department of Dentistry, UFRN, Natal, Brazil; b Postgraduate Program in Pharmaceutical Science, Federal University of Rio Grande do Norte, Natal, Brazil; c Post-Graduation program in Functional and Structural Biology/Post-graduation program Health Science/Department of Morphology, UFRN, Natal, Brazil; d Department of Dentistry, UFRN, Natal, Brazil; e Drug Quality Control Laboratory (LCQMed), Department of Pharmacy, UFRN, Natal, Brazil; f Institute of Chemistry, UFRN, Natal, Brazil; g Post-Graduation Program in Biochemistry and Molecular Biology/Post-Graduation Program in Pharmaceutical Science, Department of Biophysics and Pharmacology, UFRN, Natal, Brazil; h Post-Graduation Program in Biochemistry and Molecular Biology/Post-Graduation Program in RENORBIO, Department of Biophysics and Pharmacology, UFRN, Natal, Brazil; i Postgraduate Program in Pharmacology, Postgraduate Program in Morphology, Department of Morphology, UFC, Fortaleza, Brazil; j Postgraduate Program in Morphology, Department of Morphology, UFC, Fortaleza, Brazil; k Post-Graduation Program Oral Science/Post-Graduation Program in Pharmaceutical Science, Department of Biophysics and Pharmacology, UFRN, Natal, Brazil

**Keywords:** Polylactic-co-glycolic acid, bioavailability, diabetes

## Abstract

**Context:**

Metformin is an important oral anti-hyperglycemic used in diabetes. Polylactic-co-glycolic acid (PLGA) has been widely used due to its reliability in controlling the release of drugs.

**Objective:**

This study evaluates the *in vitro-in vivo* availability of metformin hydrochloride-loaded polylactic-co-glycolic acid.

**Material and methods:**

*In vitro* metformin release (Met-free or PLGA + Met-12.5 mg/mL per 360 min) was evaluated using static Franz vertical diffusion cells. The *in vivo* study was performed with two control groups (validation bioanalytical method) and two experimental groups of diabetic male Wistar rats treated with PLGA + Met 10 mg/kg or Met 100 mg/kg by oral gavage. Diabetes was induced by streptozotocin (40 mg/kg) through the penile vein. Blood samples were collected 0.5, 1, 4, 7, 10, 12, 18, 24, 36, 48 and 72 h and analysed by high performance liquid chromatography-tandem mass spectrometry (HPLC-MS/MS).

**Results:**

PLGA + Met 10 mg/kg was released in the *in vitro* assay suggesting a parabolic diffusion kinetic model (K −0.0619^−0.5h^) with a 100% release profile in 10 h by controlled diffusion. The *in vivo* assay showed the apparent volume of distribution Vz/F (PLGA + Met 10 mg/kg, 40971.8 mL/kg vs. Met 100 mg/kg, 2174.58 mL/kg) and mean residence time MRTinf (PLGA + Met 10 mg/kg, 37.66 h vs. Met 100 mg/kg, 3.34 h).

**Discussion and Conclusions:**

The formulation modifies pharmacokinetics parameters such as apparent distribution volume and mean residence time. The PLGA + Met 10 mg/kg had a slower elimination rate compared to Met 100 mg/kg in diabetic rats in a periodontal disease experimental model.

## Introduction

We have previously demonstrated that the association of metformin hydrochloride-with polylactic-co-glycolic acid (PLGA) was able to reduce the glucose levels and prevent inflammation and bone loss in a ligature-induced periodontitis in diabetic rats, clearly demonstrating that incorporation of metformin into PLGA improves the drug efficacy (Pereira et al. 2018). In fact, conventional drug delivery systems face limitations due to the immediate release of the drug, which hinders sustained clinical efficacy (Mourão et al. 2010). The rational development of nanoscale delivery systems for targeted and controlled drug delivery has been described in the literature (Cafferata et al. 2019). Polymeric nanoparticles (NPs) have specific advantages, due to their size and varied possibilities for surface modifications, allowing the targeted delivery of drugs to practically any compartment of the body at cellular and even sub-cellular levels (Bobrovnikova-Marjon and Hurov 2014).

Increasing the drug concentration at the action site, decreasing the concentration at the non-target sites, and consequently decreasing the undesirable effects, in addition to improving therapeutic potential are the main goals of new drug delivery systems (Sharma et al. 2012; Taghipour et al. 2018).

Among the polymers studied for nanoparticle preparation, PLGA has been widely used in several studies, due to its reliability in controlling the release of drugs for periods which can vary from days to weeks (Zhou et al. 2012). Accordingly, our previous study demonstrated that a PLGA-based drug delivery system clearly improved the effectiveness of metformin in preventing inflammation and bone loss associated with periodontal disease in diabetic rats (Pereira et al. 2018).

Several studies have described the pre-clinical efficacy of metformin encapsulated PLGA nanoparticles (Gundogdu and Cetin 2014; Lee et al. 2014). A study of *in vitro* release characteristics of chitosan-poly (lactide-co-glycolide) (CS-PLGA) nanoparticles containing metformin HCl obtained the following results: ∼20% of metformin HCL was released within 30 min and approximately 98% of the loaded metformin HCl was released at 144 h in a phosphate buffer (Gundogdu and Cetin 2014). Murphy et al. (2012) demonstrated the influence of PLGA on the drug release behaviour of metformin hydrochloride.

There are reports demonstrating the potential of nanocarriers to improve current treatment options for periodontal disease (Cafferata et al. 2019). However, their pharmacokinetic profiles are not yet well defined. In addition, as far as we know, there is no study investigating the *in vitro-in vivo* release profile of metformin hydrochloride-PLGA, orally administered to achieve systemic effects, or using a high-performance liquid chromatography-tandem mass spectrometry (HPLC-MS/MS) analytical method. Lee et al. (2014) used an elution method and a HPLC assay to characterise the *in vivo* and *in vitro* release rates of metformin from membranes used for repairing wounds associated with diabetics.

Thus, this study evaluates the *in vitro-in vivo* availability of metformin hydrochloride-loaded PLGA.

## Materials and methods

### Synthesis and characterisation of MET-loaded PLGA nanoparticles

The drug metformin hydrochloride was purchased from the Companhia da Fórmula (Brazil), PLGA 50:50 (inherent viscosity of 0.63 dL/g at 30 °C) was purchased from Birmingham Polymers Inc. (USA), polyvinyl alcohol (PVA) was purchased from Sigma-Aldrich Co. (St. Louis, MO, USA), and dichloromethane (DCM) from QHEMIS^®^ (Brazil). Purified water (0.00013 S/m) used with reverse osmosis purification equipment (OS50 LX Gehaka, São Paulo, Brazil).

MET-loaded PLGA nanoparticles were fabricated by double emulsion solvent diffusion method: 50 mg of PLGA was dissolved in 6 mL of dichloromethane (DCM). Metformin (272 mg/mL) was dissolved in an aqueous phase containing 0.1% polyvinyl alcohol. The aqueous phase with the drug (600 µL) was added into 3 mL of organic phase containing PLGA. The mixture was emulsified with a probe-tip sonicator (probe-tip diameter: 1.3 cm, Sonics & Materials Inc., Danbury, CT, USA) operating at 50% amplitude intensity for 1 min. This first mixture was then added into 6 mL of water containing 1.0% of PVA and the mixture was emulsified with a probe-tip sonicator for 1 min. This emulsion was added into 8 mL of water containing 1.0% PVA under magnetic stirring, leading to the formation of a Water/Oil/Water (W/O/W) emulsion with MET-loaded PLGA nanoparticles. The organic solvent was evaporated overnight by magnetic stirring. Free-drug nanoparticles were prepared using the same procedure, but excluding the drug.

After producing the nanoparticles, the mean diameter and particle size distribution measurements were assessed by dynamic light scattering in a ZetaPlus device (Brookhaven Instruments Co., New York, NY, USA) equipped with a 90Plus/BI-MAS apparatus at a wavelength of 659 nm, with a scattering angle of 90°. Z potential of the particles was measured by laser Doppler anemometry using the same equipment. Experimental values were given as the mean ± SD for the experiments and carried out in triplicate for each sample.

The drug loading efficiency was performed by an indirect method, in which dispersions were centrifuged at 16,900 Relative Centrifugal Force RCF (*g*) per 60 min at 4 °C using an ultra-centrifugal filter (Sartorius®, Vivaspin 2, Ultra-15 MWCO 10 kDa). The supernatant was removed and diluted in purified water 1:20 (v/v) and the measurements were carried out in a UV Thermo Fisher Scientific 60S Evolution Spectrophotometer (Waltham, MA, USA), using previously validated UV spectrophotometry at 232 nm. Entrapment efficiency (EE) was calculated using the following equation: EE% = (total drug-drug determined in the supernatant)/total drug × 100.

### *In vitro* MET release assay

*In vitro* drug release was evaluated using static Franz vertical diffusion cells, maintained at 37 ± 0.5 °C. Colloidal dispersions (2 mL) were added to the donor compartments, which were hermetically sealed and separated from the receptor compartments by 0.45 μm synthetic cellulose acetate filters that had been previously hydrated in phosphate buffer for 24 h. The receptor compartments contained 11.0 mL of phosphate buffer solution adjusted to pH 7.4 and were magnetically stirred at 360 rpm during all experiments. Next, 1.0 mL aliquots were analysed at specific time intervals by UV-Vis spectrophotometry at 232 nm. The same volume of fresh buffered solution was added back to the vessels to maintain sink conditions.

The following formulations were used in the *in vitro* release assay for 360 min:

- Met 12.5 mg/mL (Met-free)
- PLGA + Met 12.5 mg/mL (MET-loaded NP/PLGA)

All analyses were performed in triplicate; experimental values are expressed as the mean ± SD.

### *In vivo* study

#### Animals

The *in vivo* bioavailability was investigated in diabetic rats submitted to experimental periodontal disease. The experimental protocol followed the ARRIVE guidelines for animal research suggested by the National Centre for the Replacement, Refinement, and Reduction for Animals in Research. The experiments were performed on male Wistar rats (180–220 g) housed under standard conditions (12 h light/dark, 22 ± 0.1 °C), with *ad libitum* access to food and water. All animal protocols were approved by the Animal Ethics Committee of the Federal University of Rio Grande do Norte (CEUA, Protocol No 057.046/2017). The anaesthesia used to induce periodontal disease by intraperitoneal administration was Ketamine 10% (70 mg/kg, Vetnil, São Paulo, Brazil) and 2% xylazine (10 mg/kg, São Paulo, Brazil). The animals were euthanized with 80 mg/kg thiopental (Cristália, São Paulo, Brazil).

The drug concentrations were selected based on our previous study demonstrating that the association of metformin hydrochloride-with PLGA was able to reduce the glucose levels and prevent inflammation and bone loss in a ligature-induced periodontitis in diabetic rats (Pereira et al. 2018). The study was performed with two control groups (validation bioanalytical method) and two experimental groups (bioavailability of metformin loaded in PLGA microparticles and in free form).

The control groups were set to evaluate the matrix effect and selectivity of analytical method during validation procedure. It is important to make sure that no variables are able to interfere in quantifying metformin (either metformin or internal standard, isoniazid).

### Control groups for bioanalytical methodology development

A total of 4 animals were used in the control groups from which white plasma was obtained to develop the bioanalytical methodology.

The 02 control groups were:

1. Group without metformin or PLGA;
2. Group without metformin and only PLGA.

### Experimental groups for bioavailability study

The following experimental groups were formed with 4 animals per group:

1. Diabetic animals submitted to periodontal disease and treated with a single dose of 100 mg/kg/day of metformin (Met 100 mg/kg), by oral gavage;
2. Diabetic animals submitted to periodontal disease and treated with a single dose of 10 mg/kg/day of metformin + PLGA (PLGA + Met 10 mg/kg^-^), by oral gavage.

Diabetes was induced by administering streptozotocin/STZ (Sigma-Aldrisch) (40 mg/kg) through the penile vein, dissolved in sodium citrate buffer (0.01 M, pH 4.5) at the concentration of 40 mg/kg body weight under general anaesthesia with 3% isoflurane inhalation. Glucose was measured by a glycosometer (one touch select simple) after one week of STZ administration. The animals were considered diabetic upon reaching plasma glucose stability of ≥300 mg/dL, and selected for later periodontal disease studies. A puncture was made in the initial portion of the animal’s tail using a sterile needle and the blood was collected on a reagent strip for glucose determination.

After diabetes confirmation, periodontal disease induction was performed by placing a 3.0 nylon wire on the second left molar of male Wistar rats with the animals under i.p. ketamine (80 mg/kg) and xylazine (10 mg/kg) anaesthesia. Oral treatments (Met 100 mg/kg or PLGA + Met 10 mg/kg) were performed by gavage on the 10^th^ day after periodontal disease induction with a single dose. Blood collections were performed by puncturing the caudal vein at different intervals after administration: 0.5, 1, 4, 7, 10, 12, 18 and 24 h. Additional blood collections were performed in the PLGA + Met 10 mg/kg^-^ group at 36, 48 and 72 h. After collection in heparinised tubes, the blood was centrifuged 10000 rpm for 5 min at 4 °C (Refrigerated Microcentrifuge NOVATÉCNICA NT 805, 20935) and the obtained plasma (150 µL) was kept at −80 °C until analysis. The samples were prepared in a microtube (2.0 mL) by adding 50 µL plasma, 30 µL internal standard (isoniazid 10 µg/mL) and methanol to complete 300 µL (Table 1).

**Table 1.** MS/MS settings for compound-dependent parameters for metformin and internal standard isoniazid.

	Metformin	Isoniazid (IS)
Q1 mass [M + H]^+^	130.1	138.1
Q3 mass/product ion transitions *(m/z)*	60.1	121.1
Qualifier product ion transitions *(m/z)*	70.9	79.0
Declustering potential (V)	26.0	31.0
Entrance potential (V)	4.5	7.5
Collision energy (V)	17.0	15.0
Collision cell exit potential (V)	4.0	4.0

The metformin solutions to perform the calibration curve were prepared adjusting the solvent volume according to standard stock solution added to keep the 300 µL as the final volume. Then, the solutions were mixed for 1 min and centrifuged, 14000 rpm for 10 min. The supernatant was separated and analysed by HPLC-MS/MS.

### HPLC-MS/MS

Sample analysis was performed using a Dionex Ultimate 3000 liquid chromatograph system coupled to an AB Sciex QTrap 3200, triple quadrupole mass spectrometer equipped with an electrospray ionisation (ESI) Turboionspray source. The analytes (Metformin as MET and Isoniazid as INH) were chromatographically separated using an Agilent Poreshell 120 EC-C18 column (50 mm × 4.6 mm i.d., 2.7 μm) analytical column at 30 °C and an isocratic mobile phase with a flow rate set at 500 μL/min. The mobile phase consisted of (A) water and (B) acetonitrile, both containing 0.1% formic acid. The auto-sampler was maintained at 5 °C, and 10 μL of sample was injected onto the column with a total LC run time of 5 min at 4% mobile phase B. The retention times were 2.26 and 2.32 min for MET and INH (IS) respectively. The sample was analysed using multiple reaction monitoring (MRM) at unit resolution, in positive scan mode, set to detect parent [M + H]^+^ → product ion transitions for MET (*m/z* 130.1 → *m/z* 60.1) and INH (*m/z* 138.1 → *m/z* 121.1). In addition to these quantifier mass transitions, product ions of *m/z* 70.9, and 79.0 were also monitored to definitively identify MET and INH, respectively. The MS/MS specific parameters were: declustering potential 26.0 V (MET) and 31.0 V (INH), entrance potential 4.5 V (MET) and 7.5 V (INH), collision energy 17.0 V (MET) and 15.0 V (INH) and collision cell exit potential 4.0 V (MET and INH). The curtain gas was set at 18 psi and the collision gas (CAD) was set at medium to achieve optimal analyte ion resolution and fragmentation. Optimised source dependent parameters were set as follows: ion spray voltage (ISV) of 5500 V; temperature 500 °C; gas 1 (N_2_) and gas 2 (N_2_) at 45 and 40 psi, respectively. Instrument control, data acquisition and processing were carried out using Analyst, 1.5 and Chromeleon software programs via the Dionex Chromatography MS Link platform.

Each concentration was analysed in quintuplicate to build the calibration curve. The standard solutions were prepared from three different metformin stock solutions: 0.25, 1.00 and 5.00 µg/mL. The final concentrations of each point on the calibration curve were 10, 25, 50, 100, 250, 750, 1000 ng/mL. Then 30 µL of internal standard stock solution were added during the preparation of these calibration curve solutions (final isoniazid concentration, 1000 ng/mL) and the final volume of each solution was 300 µL. The lower and higher concentrations were considered as the LLQ and HLQ (lower and higher limit of quantification). The solutions of 25, 250, 750 ng/mL were prepared and considered as a low, medium and high concentration quality control samples (LQC, MQC and HQC). A dilution quality control solution (DQC) was prepared from a stock solution of 5000 ng/mL and diluted 8 times to reach the final concentration of 100 ng/mL. In addition, a 50 ng/mL solution was prepared from a 250 ng/mL solution by stock solution addition and was considered as an addition quality control (AQC). The selectivity was evaluated for 4 normal distinct plasma samples, 1 hemolyzed plasma sample and 1 lipidemic plasma sample. The plasma was submitted to the sample preparation process and the chromatographic results were compared to obtain the LLQ and no peak could have a retention time lower than 20% from MET and 5% from INH retention times. A blank plasma was injected twice prior to sample analysis to obtain the calibration curve and once after the last replicate of HLQ to perform the carry-over effect analysis. The evaluation parameters were the same as the selectivity assay. Next, the MET standard and IS were added in samples from distinct sources of 8 normal, 2 lipidemic and 2 hemolyzed plasma samples in LQC and HQC concentrations to perform the matrix effect test. The matrix effect of the PLGA microparticle solution was also evaluated. A PLGA solution without metformin (1000 ng/mL) was analysed by the same developed method and compared to blank plasma, as well to PLGA solution added to blank plasma (PLGA solution 1000 ng/mL ìn plasma). All of these parameters used in the bioanalytical method development were based on the Brazilian regulatory guide which is similar to International Council for Harmonisation of Technical Requirements for Pharmaceuticals for Human Use (ICH) protocol (Agência Nacional de Vigilância Sanitária (ANVISA) 2012; The International Council for Harmonisation of Technical Requirements for Pharmaceuticals for Human Use (ICH) 2019).

### Data analysis

The Kolmogorov-Smirnov analysis showed the independence, normality and homogeneity of variance of the residuals. The nanoparticle characterisation, pairwise comparisons of the analytical data were performed using the Student’s *t*-test. One-way analysis of variance (ANOVA) was applied for multiple comparisons, followed by Tukey’s *post hoc* test. *p* < 0.05 was considered statistically significant. Data for the *in vivo* experiments were analysed using descriptive and analytical statistics. Parametric tests such as ANOVA, followed by Bonferroni’s post-test and non-parametric Kruskall-wallis test were used. A significance level of 5% was considered. To process and analyse the data for bioavailability determination the software PKanalix version 2019R2 was used (Monolix v. 2019R1, Lixoft SAS, Antony, France, 2019). The pharmacokinetic parameters were calculated to non-compartmental analysis (NCA) model with adjusted R2 for the estimation of λz (slope of the terminal elimination phase) and linear trapezoidal integral method.

## Results

### MET-loaded PLGA nanoparticles performance

**Figure 1.**
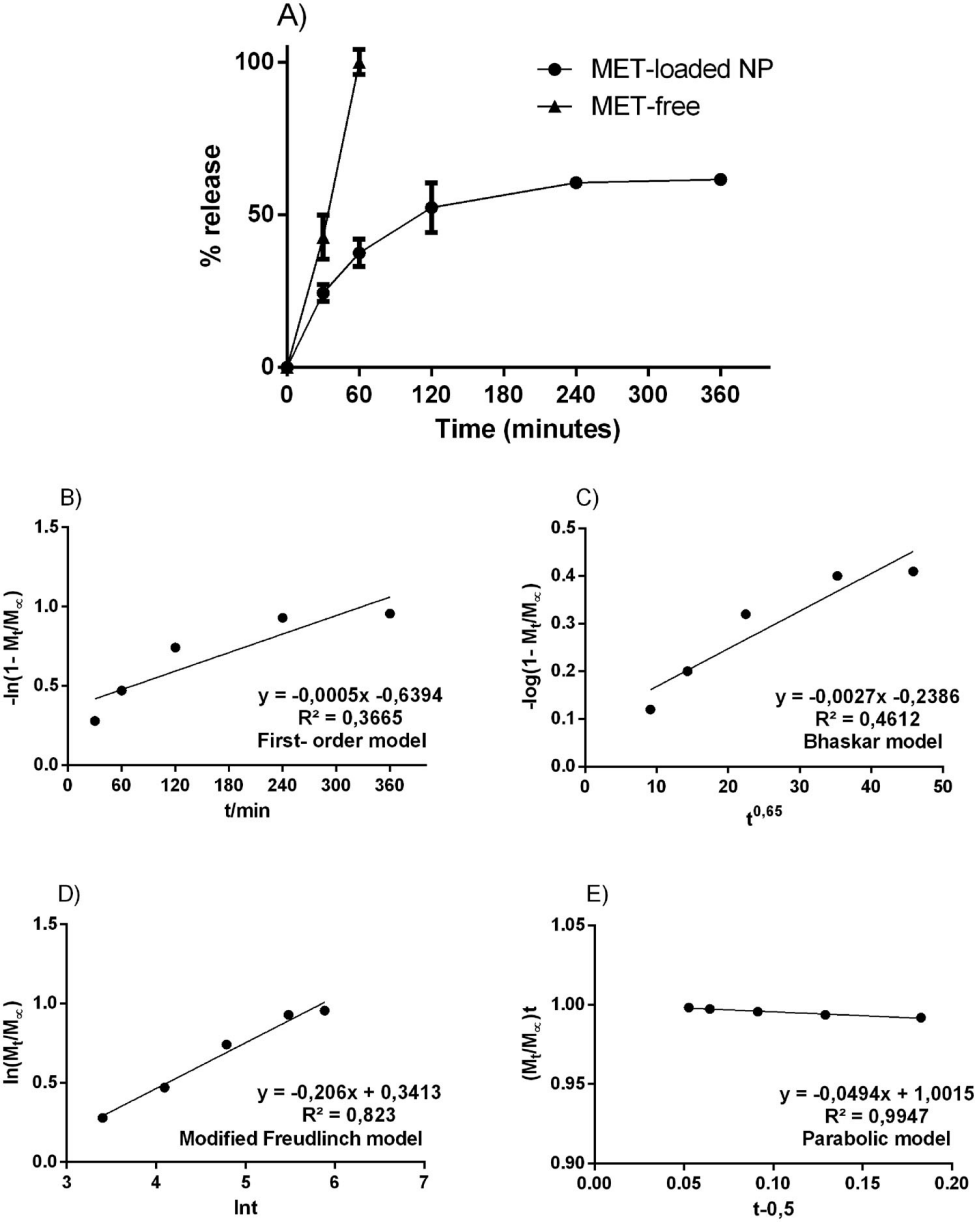
Experimental *in vitro* drug release profile from free-drug and MET-loaded nanoparticles; respective mathematical modelling adjustment of data using: B) first-order model; C) Bhaskar model; D) modified Freundlich model; and E) Parabolic model. Notes: Notes: The samples can be identified as follows: (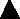) Solution of pure MET; (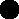) MET-loaded nanoparticles. The data are expressed as the mean ± standard deviation (SD) (*n* = 3).

The particulate system obtained an average diameter of 450.2 ± 43.4 nm, polydispersion index of 0.225 ± 0.1, zeta potential of 4.2 ± 2.4, containing encapsulation percentage of about 65% (± 4.2). After characterisation, the metformin-containing nanosystem was subjected to an *in vitro* release assay, under controlled conditions for 360 min inside the Franz Cells device. Free metformin and metformin incorporated in the nanoparticles were tested. Figure 1 shows that in the 1^st^ h of the test, metformin is completely available within the receptor compartment of the Franz cell. On the other hand, the concentration of metformin in the nanoparticles reaches 50% of its content in 2 h and remains in constant release around 60% until the end of 6 h. After that time, the systems go into exhaustion and the concentrations drop.

The data from the *in vitro* release assay, using Franz cells, were applied to suggest a possible release mechanism related to MET-loaded PLGA nanoparticles. The following kinetic models were applied (mathematical models):

- first order – ln [1- Mt/M∞] = - kt - b);
- Bhaskar – (log [1- Mt/M∞] = -Bkt0.65 + b);
- Freundlich – (ln Mt/M∞ = - klnt - b) and;
- Parabolic Diffusion –([1- Mt/M∞]/t = kt-0.5 + b).

Figure 1 also shows the kinetic constants (k) of each model and their respective correlation coefficients (r^2^). Each equation applied in the release profile represents a supposed kinetic mechanism for the drug-release. The *in vitro* profile from free-drug and MET-loaded nanoparticles is possible to notice that the parabolic diffusion model has a higher correlation coefficient (r^2^ = 0.99). The *in vitro* profile from MET-loaded nanoparticles suggested that the metformin release mechanism contained in the PLGA nanoparticles, happens by controlled diffusion of the drug release, obeying the direction from inside the particle to its surface.

### HPLC-MS/MS

**Figure 2.**
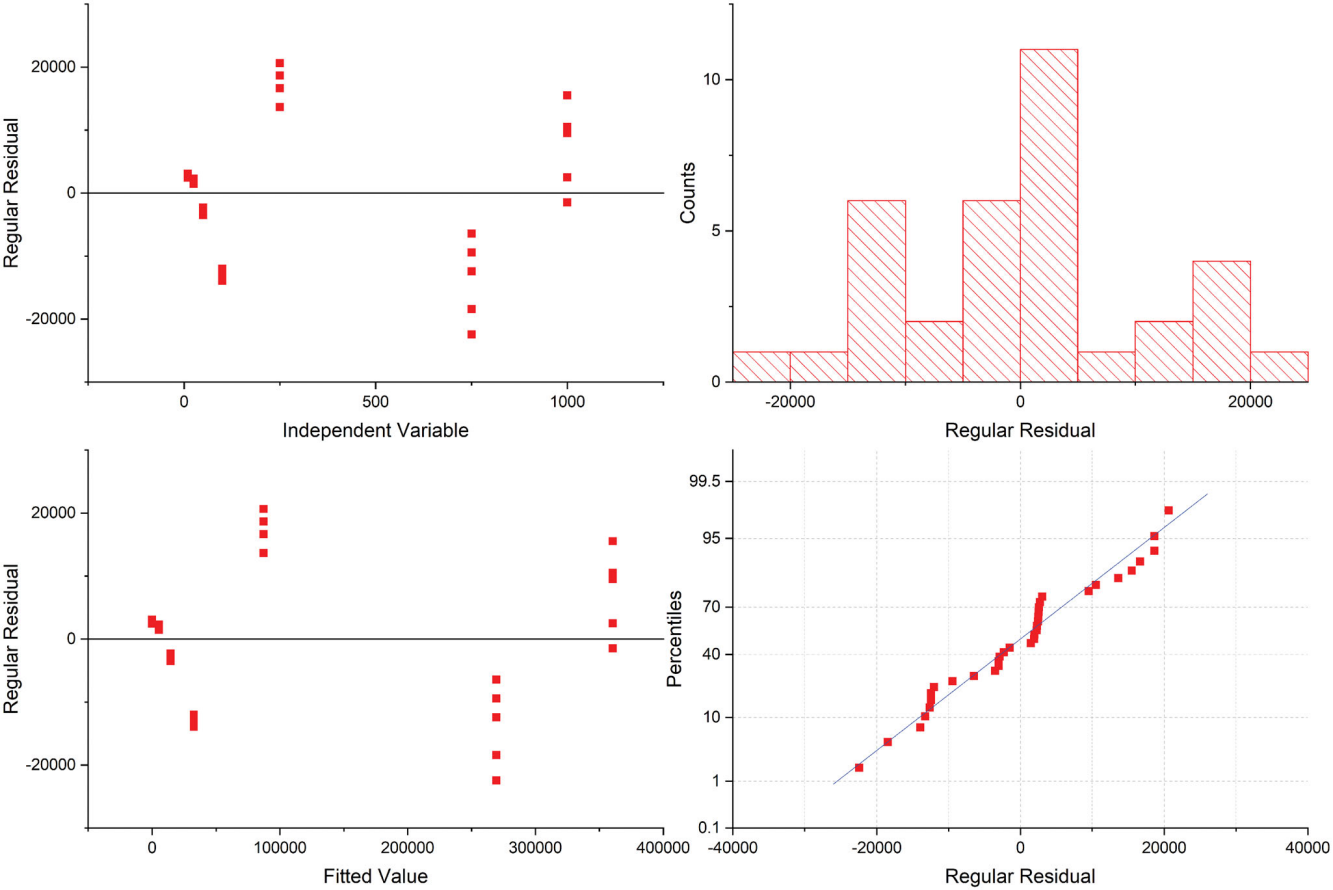
Residual plots for metformin.

Before quantifying plasma samples to obtain the bioavailability profile of metformin incorporated in the formulation containing PLGA, the HPLC-MS/MS method was developed. This step is crucial for bioanalytical quantification, which must be fast and reliable (Hopfgartner 2020). The analytical procedure developed for the determination of MET in the plasma was validated in accordance with The International Council for Harmonisation of Technical Requirements for Pharmaceuticals for Human Use (ICH) 2019. The residuals variance showed no tendency (Figure 2). Therefore, it was homogeneous for MET over the entire concentration range. Furthermore, it was observed a correlation between the peak areas and MET concentration with coefficient of determination > 0.99. The lack-of-fit was statistically significant (*p* < 0.05). However, this can be due to a large range of MET concentrations (from 10 up to 1000 ηg/mL) which does not affect the good linearity observed, according to other cited parameters. A simple sample preparation with a regular accessible commercial drug as internal standard applied to a short method that uses routine mobile phase for HPLC-MS/MS was evaluated in our study that may be applied for other HPLC detectors. A calibration curve was constructed and, according to the results, a linear correlation was found between the peak areas and MET concentration, with coefficient of determination and correlation 0.9935 and 0.9968, respectively. The intercept (a) was −3700.6 (standard deviation, s, 2463.66) and slope (b) 364.18, Table 2.

**Table 2.** Linear regression data.

Parameter	Value
Intercept, (a)	−3700.60
Slope, (b)	364.18
Standard deviation of the intercept, s(a)	2463.66
Coefficient of determination, $R^{2}$	0.9935
Coefficient of correlation, R	0.9968
Linear range (ng.mL^−1^)	10–1000

**Figure 3.**
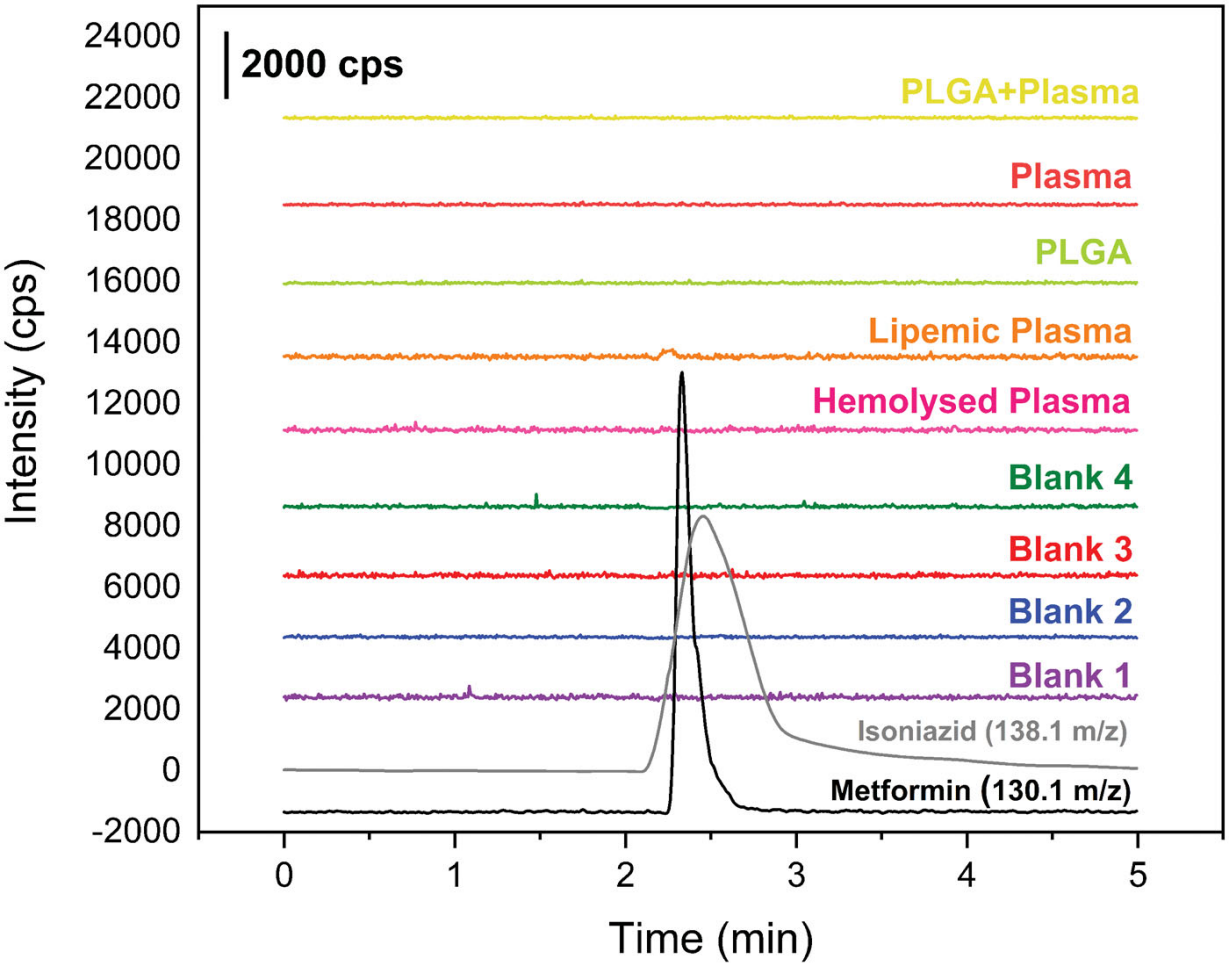
Chromatograms from selectivity study. Blank plasma chromatograms in purple, blue, red and green; blank hemolyzed plasma chromatogram in pink; blank lipemic plasma chromatogram in orange; PLGA + plasma in yellow; metformin 250 ug mL^−1^ in black (*m/z* 130.1 → *m/z* 60.1) and isoniazide 1000 ng mL^−1^ in grey (*m/z* 138.1 → *m/z* 121.1).

Other peaks were not found in the time range of MET or INH retention, as shown in Figure 3. For carryover test, no evidence of residual metformin or isoniazid after the batch analysis. It was not found any higher peak than 20% of LIQ for MET and 5% for INH. The matrix effect shows no variance higher than 15%, Table 1, satisfying the requirements. Furthermore, in both concentrations, LQC and HQC, the hemolyzed samples reach the requirements, as well as the lipemic samples. These data are summarised in Table 3.

**Table 3.** Concentration of standards used for the calibration curve.

Samples^a^	Metformin Concentration (ng.mL^−1^)	Isoniazid Concentration (ng.mL^−1^)	Final Volume (µL)
LLQ	10	1000	300
LQC	25		
AQC	50		
DQC	100		
MQC	250		
HQC	750		
HLQ	1000		

^a^LLQ: Lower Limit of Quantification; LQC: Low Quantification Control; AC: Addition Control; DQC: Diluted Quantification Control; MQC: Medium Quantification Control; High Concentration Control; HLQ: Higher Limit of Quantification.

Hence, the system has been proven to be suitable to quantify MET and provide the data about bioavailability.

### Bioavailability

**Figure 4.**
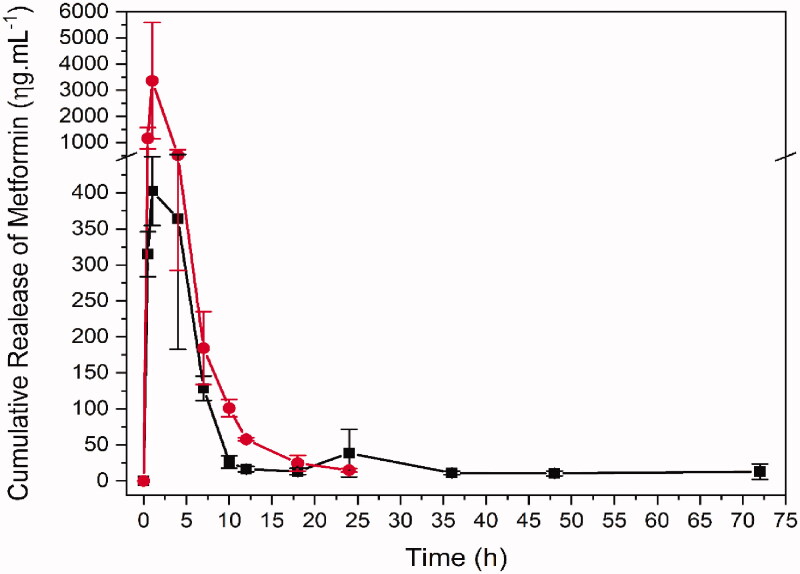
Bioavailability of metformin in single dose administration of metformin in saline and in formulation by oral gavage. Red: Met 100 mg + kg (metformin 100 mg/kg in saline solution, *n* = 4). Black: PLGA + MET 10 mg/kg (metformin 10 mg/kg in formulation containing PLGA, *n* = 4) in black.

The plasma sample quantification enabled to obtain the bioavailability profile for free and formulated Met (Figure 4), as well as to calculate the pharmacokinetic parameters for each group (Table 2). The formulation containing PLGA modified many pharmacokinetic parameters of metformin when compared to free form (metformin). As the doses are different and lower in formulation (metformin 100 mg/kg in saline solution and metformin 10 mg/kg in formulation containing PLGA), the bioavailability graphic was scaled to facilitate the comparison of profiles. Differences in metformin concentrations may be an explanation for lower Cmax and other parameters that depend on the dosage and concentration values, such as area under the curve (AUC).

In addition to bioavailability, many other pharmacokinetic parameters were remarkably changed by the formulation specially those relate to elimination, e.g., λz 0.0208 and 0.155/h, MRTinf 37.66 and 3.34 h, Cl/F 792.64 and 330.87 mL/h, Vz/F, 40971.8 and 2174.58 mL/kg for PLGA + MET 10 mg/kg and Met 100 mg/kg, respectively. Although the clearance (Cl/F) increased for metformin nanoparticles, the increase was not in the same proportion as apparent volume of distribution (Vz/F) and, consequently, the elimination rate was lower. The λz, Vz/F and MRT show this slower elimination rate for the formulation group, corroborating the observation in the graphic and parameters present in the Table 2.

Before plasma sample quantification to obtain bioavailability profile of metformin incorporated in formulation containing PLGA, an HPLC-MS/MS method was developed. A calibration curve was constructed and, according to results, a linear correlation was found between the peak areas and MET concentration, with coefficient of determination > 0.99. Detailed results are shown in Tables 2 and 3. The selectivity results were satisfactory. Other peaks were not found in the time range of MET or INH retention, as shown in Figure 2. For carry over test, no evidence of residual metformin or isoniazid after the batch analysis. No peaks were found higher than 20% of LIQ for MET and 5% for INH. The matrix effect shows no variance higher than 15%, satisfying the requirements. Furthermore, in both concentrations, LQC and HQC, the hemolyzed samples reach the requirements, as well as the lipemic samples. Also, the PLGA placebo attends the applications for matrix effect. These data are summarised in Table 4. Hence, the system was suitable to quantify MET and provide the data about bioavailability.

**Table 4.** Matrix effect results.

Samples	Concentration (ng.mL^−1^)	NMF*	CV (%)
Blank 1	25.9	1.04	0.58
Blank 2	34	1.36	0.44
Blank 3	31.2	1.25	0.48
Blank 4	24.7	0.99	0.60
Lipemic 1	24.2	1.02	0.62
Lipemic 2	25.5	1.02	0.59
Hemolyzed 1	27.1	1.08	0.55
Hemolyzed 1	23.2	0.93	0.64
Blank 5	815	1.09	0.02
Blank 6	907	1.21	0.02
Blank 7	930	1.24	0.02
Blank 8	890	1.19	0.02
Lipemic 3	1010	1.35	0.02
Lipemic 4	1280	1.71	0.01
Hemolyzed 5	985	1.31	0.02
Hemolyzed 6	993	1.32	0.02
PLGA	10.2	0.98	0.05
Plasma	10.1	1.11	0.01
PLGA + Plasma	10.2	1.07	0.08

*NMF: Non-negative matrix factorization.

The plasma sample quantification enabled us to obtain the bioavailability profile for Met 100 mg/kg or PLGA + MET 10 mg/kg (Figure 3), as well as to calculate the pharmacokinetic parameters for each group (Table 5).

**Table 5.** Parameters estimates of MET pharmacokinetics with noncompartmental model in rat plasma by PKanalix version 2019R2 for PLGA + MET 10 mg kg^−1^ (metformin 10 mg kg^−1^ in formulation containing PLGA, *n* = 4) and MET 100 mg kg^−1^ (metformin 100 mg Kg^−1^ in saline solution, *n* = 4).

Pharmacokinetic parameter	PLGA + MET 10mg kg^−1^	MET 100 mg kg^−1^
	Mean (*n* = 4)	Mean (*n* = 4)
AUC_0→∞_ (ng . mL^−1^ . h)	4374.41 ± 1636.91	9325.81 ± 3777.56
%AUC_extrap_	20.46 ± 11.76	1.22 ± 0.605
AUC_0→t_ (ng . mL^−1^ . h)	3370.14 ± 800.35	9227.35 ± 3781.95
AUClast (ng . mL^−1^ . h)	3343.35 ± 834.5	9227.35 ± 3781.95
AUMC_0→∞_ (ng . mL^−1^ . h^2^)	190855.35 ± 158974.35	28231.31 ± 3250.28
AUMClast (ng . mL^−1^ . h^2^)	42608.59 ± 19810.43	25200.59 ± 3262.49
Cl/F (mL . h^−1^)	0.894 ± 0.334	4.28 ± 1.76
Clast (ng . mL^−1^)	15.11 ± 7.78	14.85 ± 2.32
Cmax (ng . mL^−1^)	470.63 ± 83.98	3377.5 ± 2217.24
λz (h^−1^)	0.0208 ± 0.0116	0.155 ± 0.0273
MRTinf (h)	37.66 ± 21.84	3.34 ± 1.04
MRTlast (h)	12.24 ± 3.37	3.01 ± 0.91
Tmax (h)	2.5 ± 1.73	0.875 ± 0.25
Vz/F (mL)	46.31 ± 10.41	28.08 ± 12.56

t = last collection time (Metformin in saline 24 h and in formulation 72 h). The results are presented as value ± SD and have *p* < 0.05.

The formulation containing PLGA + MET 10 mg/kg modified many pharmacokinetic parameters of metformin when compared to Met 100 mg/kg. Differences in metformin concentrations may be an explanation for lower Cmax and other parameters that depend on the concentration values, such as area under the time curve (AUC).

In addition to bioavailability, many other pharmacokinetics parameters were remarkably changed by the formulation, specially those relate to elimination, e.g., λz 0.0208 and 0.155/h, MRTinf 37.66 and 3.34 h, Cl/F 792.64 and 330.87 mL/h, Vz/F, 40971.8 and 2174.58 mL/kg for PLGA + MET 10 mg/kg and Met 100 mg/kg, respectively. Although the Cl/F increased for metformin nanoparticles, the increase was not in the same proportion as Vz/F and, consequently, the elimination rate was lower. The λz, Vz/F and MRT show this slower elimination rate for the PLGA + MET 10 mg/kg group, corroborating the observation in the Figure 4.

## Discussion

The *in vitro* profile from MET-loaded nanoparticles suggested that the metformin release mechanism contained in the PLGA nanoparticles happens by controlled diffusion of the drug release, obeying the direction from inside the particle to its surface and mechanism involved includes the diffusion of molecules through particles and from a flat surface of nanoparticles (Fenglin et al. 2013). On the other hand, several studies show the efficacy of nanoparticle performance. However, most of these studies suggests that metformin activity is relate to pH-dependence and the nanosystems preparation have a complex synthesis process to obtain the nanoparticles (Patiño-Herrera et al. 2019; Wook Huh et al. 2021). The *in vivo* profile showed slower elimination is for metformin administrated in the formulation. The pharmacokinetic curve for PLGA + MET 10 mg/kg also indicates that within 24 h there is a tendency to increase the drug’s plasma concentration followed by reabsorption of the eliminated metformin. Associated to high Vz/F, this may indicate a delayed drug release from the PLGA. Mandl et al. (2019) suggest that size of PLGA NPs can be used to tune delivery to certain tissues and cell populations *in vivo*. However, the reabsorption of the eliminated metformin cannot be ruled out since this process is known and relevant to its pharmacokinetics (Kimura et al. 2005; Gong et al. 2012; Shibata et al. 2020). Further studies should be carried out to elucidate the mechanism involved in increasing the plasma concentration of metformin (when in association with PLGA) in the 24 h period, as well as in the higher MRT. Some studies aimed to develop metformin nanoparticles using different materials as SiO_2_, alginate and hyaluronic acid to control MET release, raise metformin MRT and also increase bioavailability (Kumar et al. 2017; Bhujbal and Dash 2018; Patiño-Herrera et al. 2019). Wang et al. (2021) used chitosan to make the MET nanoparticles for polycystic kidney disease treatment and found a higher AUC, as well as controlled release, improving the delivery of the drug accumulated in some organs, mainly in the intestine. Wook Huh et al. (2021) evaluated a hollow-core floating tablet (HCFT) based in mixture of hydroxypropyl and microcrystalline cellulose showing a higher bioavailability with the same MRT, approximately 35 h, compared to a commercial metformin tablet. Among the different materials that have been associated to MET for obtaining MET nanoparticles, only PLGA was able to sustain MET plasma concentration over 72 h, with higher AUC and Vz/F. These findings suggest that PLGA-based drug delivery system may be a promising strategy for the treatment of chronic diseases. Studies showed that MET associated with PLGA presented a therapeutic effect similar to that of the free drug, with the benefit of using a 10 times lower concentration of the drug. The association of metformin with PLGA allows its use as an adjuvant in other treatments, such as hypertension, renal disease, or lung fibrosis in diabetic patients (Gong et al. 2012; Corremans et al. 2018; Foretz et al. 2019; Han and Choi 2020). The present study demonstrates the bioavailability of metformin hydrochloride-loaded PLGA nanoparticles. The concentration evaluated in the present work was previously proven to be effective in the treatment of inflammation and bone loss associated with periodontal disease, in diabetic rats (Pereira et al. 2018). As far as we know, there is no study in the literature on in vivo bioavailability for metformin hydrochloride-loaded PLGA nanoparticles. Lee et al. (2014) have described the concentration of metformin, release from nanofibrous drug-eluting membranes, at the site of action (skin wounds associated with diabetics). Several strategies have been used to control the release of metformin for clinical applications, such as the new formulations of release and extended-release formulations of metformin. We have demonstrated in the present study, in addition to a controlled release of metformin from PLGA (as observed in the AUC), an effective metformin modified-release system. According to the literature, the dose of metformin used in rats is about 100 mg/kg, which is equivalent to an average dose between 850 to 1000 mg/day for human adults (60 kg). In the present study, the association of metformin with PLGA represents a significant reduction in the daily dose used by an adult. Similarly, we have found significant lower glycemic levels in the blood of diabetic rats submitted to periodontal disease and treated with metformin associated with PLGA (Pereira et al. 2018). In addition, we demonstrated that PLGA with a lower dose was able to sustain MET plasma concentration over 72 h.

## Conclusions

Our *in vitro* availability results suggest parabolic diffusion kinetic model with release profile 100% by 10 h by controlled diffusion of the drug release, obeying the direction from inside the particle to its surface. The present study is unique since it describes for the first time the pharmacokinetics characteristics of metformin hydrochloride associated with PLGA using HPLC-MS/MS analytical method. *In vivo* availability of metformin hydrochloride-PLGA nanoparticles in diabetic rats in a periodontal disease experimental model **s**howed that the Vz/F (PLGA + Met 10 mg/kg, 40971.8 mL/kg vs. Met 100 mg/kg 2174.58 mL/kg) and MRTinf (PLGA + Met 10 mg/kg, 37.66 h vs. Met 100 mg/kg 3.34 h) evidenced the slower elimination rate in PLGA + Met 10 mg/kg formulation.

## References

[CIT0001] Agência Nacional de Vigilância Sanitária (ANVISA) 2012. Requisitos mínimos para a validação de métodos bioanalíticos empregados em estudos com fins de registro e pós-registro de medicamentos [Minimum requirements for validation of bioanalytical methods used in studies for drug registration and post-registration purposes]. Resolution. 27:93.

[CIT0002] Bhujbal S, Dash AK. 2018. Metformin-loaded hyaluronic acid nanostructure for oral delivery. AAPS PharmSciTech. 19(6):2543–2553. 29948986 10.1208/s12249-018-1085-1

[CIT0003] Bobrovnikova-Marjon E, Hurov JB. 2014. Targeting metabolic changes in cancer: novel therapeutic approaches. Annu Rev Med. 65:157–170. 24422570 10.1146/annurev-med-092012-112344

[CIT0004] Cafferata EA, Alvarez C, Diaz KT, Maureira M, Monasterio G, González FE, Covarrubias C, Vernal R. 2019. Multifunctional nanocarriers for the treatment of periodontitis: Immunomodulatory, antimicrobial, and regenerative strategies. Oral Dis. 25(8):1866–1878. 30565778 10.1111/odi.13023

[CIT0005] Corremans R, Vervaet BA, D’Haese PC, Neven E, Verhulst A. 2018. Metformin: a candidate drug for renal diseases. IJMS. 20(1):42–56. 10.3390/ijms20010042 PMC6337137 30583483

[CIT0006] Fenglin QI, Xiaoqing Z, Shuping L. 2013. A novel method to get methotrexatum/layered double hydroxides intercalation compounds and their release properties. J Phys Chem Solids. 74(8):1101–1108.

[CIT0007] Foretz M, Guigas B, Viollet B. 2019. Understanding the glucoregulatory mechanisms of metformin in type 2 diabetes mellitus. Nat Rev Endocrinol. 15(10):569–589. 31439934 10.1038/s41574-019-0242-2

[CIT0008] Gong L, Goswami S, Giacomini KM, Altman RB, Klein TE. 2012. Metformin pathways: pharmacokinetics and pharmacodynamics. Pharmacogenet Genomics. 22(11):820–827. 22722338 10.1097/FPC.0b013e3283559b22 PMC3651676

[CIT0009] Gundogdu N, Cetin M. 2014. Chitosan-poly (lactide-co-glycolide) (CS-PLGA) nanoparticles containing metformin HCl: preparation and *in vitro* evaluation. Pak J Pharm Sci. 27(6):1923–1929. 25362616

[CIT0010] Han SY, Choi YH. 2020. Pharmacokinetic interaction between metformin and verapamil in rats: inhibition of the OCT2-mediated renal excretion of metformin by verapamil. Pharmaceutics. 12(5):468–483. 10.3390/pharmaceutics12050468 PMC7284374 32455555

[CIT0011] Hopfgartner G. 2020. Bioanalytical method validation: How much should we do and how should we document? Anal Bioanal Chem. 412(3):531–532. 31838559 10.1007/s00216-019-02334-8

[CIT0012] Kimura N, Masuda S, Tanihara Y, Ueo H, Okuda M, Katsura T, Inui K. 2005. Metformin is a superior substrate for renal organic cation transporter OCT2 rather than hepatic OCT1. Drug Metab Pharmacokinet. 20(5):379–386. 16272756 10.2133/dmpk.20.379

[CIT0013] Kumar S, Bhanjana G, Verma RK, Dhingra D, Dilbaghi N, Kim KH. 2017. Metformin-loaded alginate nanoparticles as an effective antidiabetic agent for controlled drug release. J Pharm Pharmacol. 69(2):143–150. 28033667 10.1111/jphp.12672

[CIT0014] Lee CH, Hsieh MJ, Chang SH, Lin YH, Liu SJ, Lin TY, Hung KC, Pang JH, Juang JH. 2014. Enhancement of diabetic wound repair using biodegradable nanofibrous metformin-eluting membranes: *in vitro* and *in vivo*. ACS Appl Mater Interfaces. 6(6):3979–3986. 24568239 10.1021/am405329g

[CIT0015] Mandl HK, Quijano E, Suh HW, Sparago E, Oeck S, Grun M, Glazer PM, Saltzman WM. 2019. Optimizing biodegradable nanoparticle size for tissue-specific delivery. J Control Release. 314:92–101. 31654688 10.1016/j.jconrel.2019.09.020 PMC6909251

[CIT0016] Mourão SC, da Silva C, Bresolin TM, Serra CH, Porta V. 2010. Dissolution parameters for sodium diclofenac-containing hypromellose matrix tablet. Int J Pharm. 386(1-2):201–207. 19941944 10.1016/j.ijpharm.2009.11.022

[CIT0017] Murphy C, Pillay V, Choonara YE, Du Toit LC, Ndesendo VM, Chirwa N, Kumar P. 2012. Optimization of a dual mechanism gastrofloatable and gastroadhesive delivery system for narrow absorption window drugs. AAPS Pharm Sci Tech. 13(1):1–15. 10.1208/s12249-011-9711-1 PMC3299464 22048877

[CIT0018] Patiño-Herrera R, Louvier-Hernández JF, Escamilla-Silva EM, Chaumel J, Escobedo AGP, Pérez E. 2019. Prolonged release of metformin by SiO2 nanoparticles pellets for type II diabetes control. Eur J Pharm Sci. 131:1–8. 30735819 10.1016/j.ejps.2019.02.003

[CIT0019] Pereira A, Brito G, Lima M, Silva Júnior A, Silva E, de Rezende A, Bortolin R, Galvan M, Pirih F, Araújo Júnior R, et al. 2018. Metformin hydrochloride-loaded PLGA nanoparticle in periodontal disease experimental model using diabetic rats. IJMS. 19(11):3488–3504. 10.3390/ijms19113488 PMC6274734 30404181

[CIT0020] Sharma A, Madhunapantula SV, Robertson GP. 2012. Toxicological considerations when creating nanoparticle-based drugs and drug delivery systems. Expert Opin Drug Metab Toxicol. 8(1):47–69. 22097965 10.1517/17425255.2012.637916 PMC3245366

[CIT0021] Shibata M, Toyoshima J, Kaneko Y, Oda K, Nishimura T. 2020. A drug-drug interaction study to evaluate the impact of peficitinib on OCT1- and MATE1-mediated transport of metformin in healthy volunteers. Eur J Clin Pharmacol. 76(8):1135–1141. 32472157 10.1007/s00228-020-02876-2 PMC7351850

[CIT0022] Taghipour YD, Bahramsoltani R, Marques AM, Naseri R, Rahimi R, Haratipour P, Iranpanah A, Panah AI, Farzaei MH, Abdollahi M. 2018. A systematic review of nano formulation of natural products for the treatment of inflammatory bowel disease: drug delivery and pharmacological targets. Daru. 26(2):229–239. 30382546 10.1007/s40199-018-0222-4 PMC6279665

[CIT0023] The International Council for Harmonisation of Technical Requirements for Pharmaceuticals for Human Use (ICH) 2019. Bioanalytical Method Validation M10; [accessed 2021 Apr 30]. http://ema.europa.eu/en/documents/scientific-guideline/draft-ich-guideline-m10-bioanalytical-method-validation-step-2b_en.pdf

[CIT0024] Wang J, Chin D, Poon C, Mancino V, Pham J, Li H, Ho PY, Hallows KR, Chung EJ. 2021. Oral delivery of metformin by chitosan nanoparticles for polycystic kidney disease. J Control Release. 329:1198–1209. 33127449 10.1016/j.jconrel.2020.10.047 PMC7904655

[CIT0025] Wook Huh H, Na YG, Kang H, Kim M, Han M, Mai Anh Pham T, Lee H, Baek JS, Lee HK, Cho CW. 2021. Novel self-floating tablet for enhanced oral bioavailability of metformin based on cellulose. Int J Pharm. 592:120113. 33246050 10.1016/j.ijpharm.2020.120113

[CIT0026] Zhou J, Patel TR, Fu M, Bertram JP, Saltzman WM. 2012. Octa-functional PLGA nanoparticles for targeted and efficient siRNA delivery to tumors. Biomaterials. 33(2):583–591. 22014944 10.1016/j.biomaterials.2011.09.061 PMC4204797

